# Retraction notice to “Sudip Bhattacharya. Strengthening public health surveillance through blockchain technology” [*AIMS Public Health*, 2019, 6(3): 326–333]

**DOI:** 10.3934/publichealth.2023002

**Published:** 2023-01-18

**Authors:** Sudip Bhattacharya, Amarjeet Singh, Md Mahbub Hossain

**Affiliations:** 1 Department of Community Medicine, Himalayan Institute of Medical Sciences, Dehradun, India; 2 Department of Community Medicine, School of Public Health, Postgraduate Institute of Medical Education and Research, Chandigarh, India; 3 Affiliation Department of Health Promotion and Community Health Sciences, School of Public Health, Texas A & M University, Texas, USA

The journal retracts the paper entitled “Strengthening public health surveillance through blockchain technology” [1]. This article is very similar to Chattu VK, et al. “The emerging role of blockchain technology applications in routine disease surveillance systems to strengthen global health security” [2]. In accordance with our publishing policies, an investigation was conducted that confirmed the overlap. The article [1] is therefore retracted and shall be marked accordingly.

This retraction was approved by the Editor in Chief of the journal AIMS Public Health.

The authors agreed to this retraction.

## References

[1] Bhattacharya Sudip, Singh Amarjeet, Hossain Md Mahbub (2019). Strengthening public health surveillance through blockchain technology. AIMS Public Health.

[2] Chattu VK, Nanda A, Chattu SK (2019). The emerging role of blockchain technology applications in routine disease surveillance systems to strengthen global health security. Big Data Cogn Comput.

